# Combined Effects of Dietary Gum Arabic and Mannan Oligosaccharides on Growth Performance, Physiological Responses, Meat Quality, and Selected Cecal Bacteria in Growing New Zealand White Rabbits

**DOI:** 10.3390/ani16172680

**Published:** 2026-08-27

**Authors:** Islam M. Youssef, Yasser Alrauji, Mohamed Shehab-El-Deen

**Affiliations:** 1 Animal Production Systems Research Department, Animal Production Research Institute, Agricultural Research Center, Giza 12618, Egypt; 2 Department of Animal and Poultry Production, College of Agriculture and Food, Qassim University, Buraydah 51452, Saudi Arabia; 3 Animal Production Department, Faculty of Agriculture, Suez Canal University, Ismailia 41522, Egypt

**Keywords:** rabbits, nutrition, gum Arabic, mannan oligosaccharides, antioxidants, prebiotics, microbiota

## Abstract

The interest in dietary prebiotics in rabbit production has increased as a consequence of the search for natural alternatives to antibiotic growth promoters. This study was conducted to evaluate the individual and combined effects of gum Arabic and mannan oligosaccharides on growth performance, physiological health, meat quality and cecal bacterial populations of growing New Zealand White rabbits. Eighty rabbits (5 weeks of age) were randomly allocated to four dietary treatments, for a period of 8 weeks: basal diet (control), basal diet supplemented with gum Arabic (2 g/kg), mannan oligosaccharides (2 g/kg) or their combination (2 + 2 g/kg). The dietary supplementation significantly improved the final body weight, body weight gain, feed conversion ratio, carcass yield, serum protein profile, selected serum biochemical indicators related to hepatic and renal status, lipid profile, antioxidant status, immune response, meat quality and cecal microbial balance as compared to the control group. The combined treatment always produced the best improvements with better growth performance, less abdominal fat, higher antioxidant enzyme activity, higher immunoglobulin concentrations, better water-holding capacity and cooking characteristics of meat, higher populations of beneficial *Lactobacillus* and lower *Escherichia coli* counts. The combined treatment produced the most favorable responses for several measured variables, although the magnitude and statistical significance of the response varied among traits. These findings suggest that GA and MOS may have potential as dietary supplements for modulating selected productive, physiological, meat-quality, and cecal bacterial parameters in growing rabbits.

## Simple Summary

Digestive disorders and oxidative stress are frequent in growing rabbits and may adversely affect growth and general health. As the use of antibiotic growth promoters is decreasing, the use of natural feed additives is an important alternative. This study aimed to investigate the effect of two natural prebiotics, gum Arabic (GA) and mannan oligosaccharides (MOS), either alone or in combination, on rabbit performance. Eighty New Zealand White growing rabbits were fed either a standard diet or diets supplemented with gum Arabic, mannan oligosaccharides or a combination of both for 8 weeks. The rabbits that received both supplements exhibited significant improvements in body weight, feed efficiency, carcass yield, meat quality, antioxidant status, immune response, and beneficial intestinal bacteria. They also had better blood markers for selected indicators related to hepatic and renal status and fat metabolism, and lower levels of harmful bacteria in the cecum. The combined supplementation gave better results than either additive alone and indicates that the two ingredients work together to support animal health and productivity. These results indicate that the combination of gum Arabic and mannan oligosaccharides is a promising natural nutritional strategy for producing healthier rabbits with better-quality meat.

## 1. Introduction

Rabbit production is an important component of livestock production because rabbits are fast-growing animals with high feed efficiency that produce lean, nutritious meat characterized by high-quality protein and relatively low-fat content [1,2]. Nevertheless, growth performance is frequently constrained by intestinal disorders, oxidative stress, inefficient nutrient utilization, and susceptibility to infections. Considering global efforts to eliminate routine antibiotic growth promoters due to concerns regarding antimicrobial resistance and tissue residues, there is growing interest in exploring alternative dietary strategies [3,4]. In this context, prebiotics have received attention for their ability to support beneficial gut microbiota populations, enhance nutrient absorption, reinforce intestinal barrier function, and modulate immune and antioxidant responses [5,6,7]. However, whether natural prebiotics can produce functional responses comparable to traditional dietary additives under production conditions remains to be fully established. Among the various types of prebiotics, gum Arabic (GA) and mannan oligosaccharides (MOS) are of particular interest owing to their complementary features and benefits.

Gum Arabic is a natural exudate obtained mainly from *Acacia senegal* and *Acacia seyal*, composed largely of highly branched arabinogalactan polysaccharides that resist digestion in the upper gastrointestinal tract [8]. As a fermentable soluble fiber, GA reaches the hindgut, where microbial fermentation produces short-chain fatty acids (SCFAs), including acetate, propionate, and butyrate, which support intestinal epithelial function and gut barrier integrity [9]. GA has also been associated with antioxidant and anti-inflammatory activities through modulation of pathways such as Nuclear Factor Erythroid 2-Related Factor 2 (Nrf2) and Nuclear Factor-Kappa B (NF-κB) [10]. Previous studies have reported beneficial effects of GA on growth performance, nutrient utilization, antioxidant status, and intestinal health in different animal species, although responses depend on species and supplementation level [11]. Reported dietary levels have ranged from approximately 1.2 to 60 g/kg in broilers, 10 to 70 g/kg in laying hens, 1 to 2 g/kg in growing rabbits, and 5 to 15 g/kg in rabbit does, with beneficial effects on growth, feed efficiency, egg mass, milk yield, or kit survival reported at different inclusion levels [9,10].

Another widely used prebiotic in animal nutrition is mannan oligosaccharides (MOS), which are commonly derived from the cell wall of *Saccharomyces cerevisiae*. MOS is recognized primarily for interfering with the adhesion of bacteria bearing mannose-specific type-1 fimbriae, including *Escherichia coli* and *Salmonella* spp., thereby limiting pathogen colonization and supporting intestinal microbial balance [12,13]. It has also been associated with improved mucosal immune responses and intestinal barrier function [14]. Importantly, responses to MOS vary according to dose and animal species [12,15].

Although GA and MOS have each been investigated as individual feed additives, their combined use in growing rabbits remains insufficiently studied. GA primarily provides a fermentable substrate that supports microbial fermentation and SCFA production [15,16], whereas MOS acts mainly through pathogen-adhesion interference and modulation of mucosal immunity [17]. These complementary modes of action provide a rationale for evaluating GA and MOS individually and in combination in growing rabbits, while recognizing that their responses may vary with supplementation level and production conditions.

Accordingly, the present study aimed to evaluate the effects of GA and MOS, administered individually or as a combined dietary treatment, on the productivity and physiological responses of growing New Zealand White (NZW) rabbits. The study comprehensively assessed growth performance, carcass characteristics, blood biochemical indices, antioxidant and immune responses, meat quality, and selected cecal microbial populations. The combined treatment was expected to provide favorable responses compared with the individual supplementation treatments.

## 2. Materials and Methods

The experiment was conducted at a private farm in New Salhia City, Sharqia Governorate, Egypt. The Institutional Animal Care and Use Committee of the Department of Animal Production, Faculty of Agriculture, Suez Canal University, Ismailia, Egypt, sanctioned all relevant animal experimentation protocols under No. SCU-AGR-REC 21/2026. All techniques employed were executed in accordance with the regulations and directions of the Committee.

### 2.1. Experimental Design and Animal Management

Eighty healthy New Zealand White rabbit bucks, aged 5 weeks, were individually weighed and randomly assigned to one of four dietary regimens in a completely randomized design, with 20 rabbits per treatment. The control group (T1) received the basal diet without supplementation, whereas T2 received the basal diet supplemented with 2 g/kg gum Arabic (GA), T3 received the basal diet supplemented with 2 g/kg mannan oligosaccharides (MOS), and T4 received the basal diet supplemented with GA and MOS at 2 g/kg each, resulting in a total supplementation level of 4 g/kg diet. The required amounts of GA and MOS were thoroughly mixed with the basal diet before pelleting to ensure uniform distribution of the supplements throughout the experimental diets. The inclusion level of 2 g/kg for each additive was selected based on previously reported dietary supplementation levels of GA and MOS in monogastric animals, including rabbits, and was considered a practical level for evaluating their individual and combined effects [9,17].

All rabbits exhibited clinical health, were devoid of external parasites and skin diseases, and had an initial average body weight of 776 ± 3.12 g. The feeding trial lasted for 8 weeks, from 5 to 13 weeks of age. Each rabbit was individually kept in a galvanized wire cage (50 × 50 × 40 cm) furnished with a stainless-steel feeder and an automatic nipple drinker, providing ad libitum access to feed and fresh water. During the trial, all animals were kept under uniform management and environmental circumstances. The ambient temperature varied from 20 to 30 °C, while relative humidity was sustained between 55 and 63%. A lighting regimen of 16 h of illumination and 8 h of darkness was implemented over the study duration.

The basal diet was supplied in pelleted form and was formulated to meet the nutritional requirements of growing rabbits according to the National Research Council [18]. The diet consisted of alfalfa hay (26.5%), barley (17.0%), yellow corn (15.0%), soybean meal (44%; 16.0%), wheat bran (20.0%), alfalfa straw (3.0%), limestone (1.65%), a vitamin-mineral premix (0.30%), NaCl (0.30%), DL-methionine (0.10%), an anticoccidial (0.05%), and an antitoxin (0.10%). The calculated chemical composition of the basal diet was 17.5% crude protein, 2.8% fat, 10.0% crude fiber, and 2600 kcal/kg of digestible energy (equivalent to approximately 10.88 MJ/kg). The complete ingredient and calculated chemical composition of the basal diet are presented in Table 1. The basal diet presented in Table 1 represents the complete unsupplemented diet and totals 100%. Gum Arabic (GA) and mannan oligosaccharides (MOS) were added to the basal diet as supplemental ingredients and did not replace any ingredient listed in Table 1. The additives were thoroughly mixed with the basal diet before pelleting to ensure uniform distribution.

### 2.2. Data Collection

#### 2.2.1. Growth Performance

Throughout the experimental period, each rabbit was weighed individually on a weekly basis to the nearest gram. Total body weight gain (TBWG) was calculated at the end of the experimental period as the difference between final body weight at 13 weeks of age and initial body weight at 5 weeks of age. Total feed intake (FI) was determined for the entire experimental period (from 5 to 13 weeks of age) for each rabbit by subtracting the total amount of feed remaining at the end of the experimental period from the total amount of feed offered throughout the experimental period and was expressed as g/rabbit. Feed conversion ratio (FCR) was calculated as the total amount of feed consumed (g) divided by the corresponding total body weight gain (g) during the entire experimental period.

#### 2.2.2. Carcass Features

At the conclusion of the experimental period (13 weeks of age), seven rabbits per treatment were randomly selected from the 20 rabbits assigned to each treatment for slaughter-related measurements, including carcass characteristics, serum biochemical analyses, antioxidant and immune measurements, meat quality, and cecal bacterial enumeration. The slaughter subset was selected to provide biological replicates across treatment groups while limiting the number of animals subjected to terminal sampling. After a 12-h feed withdrawal, with unrestricted access to water, rabbits were weighed individually on the morning of slaughter to ascertain their pre-slaughter body weight. Rabbits were humanely slaughtered in accordance with Islamic slaughtering principles by severing the jugular veins and carotid arteries using a sharp, sterile knife. The animals were then allowed to undergo complete exsanguination before further processing and carcass evaluation. Subsequent to exsanguination, the carcasses were weighed, skinned, and eviscerated. The weights of the carcass, consumable giblets (liver, heart, and kidneys), and visceral fat were documented. All carcass characteristics were represented as a proportion of the pre-slaughter body mass.

#### 2.2.3. Blood Biochemical Parameters

Blood samples were obtained from the same seven rabbits per treatment immediately post-slaughter after an overnight fast. Samples were placed in non-heparinized tubes, permitted to clot, then centrifuged at 3000 rpm for 15 min to isolate the serum. The collected serum was preserved at −20 °C until biochemical analysis was conducted. Total protein (TP) and albumin (ALB) serum concentrations were measured with commercial diagnostic kits following the methodology outlined by Henry et al. [19]. The concentration of serum globulin (GLOB) was determined by deducting albumin from total protein. Serum alanine aminotransferase (ALT) and aspartate aminotransferase (AST) activity were quantified with commercial reagent kits from Reactivos GPL CHEMELEX, S.A., Canovelles, Barcelona, Spain. Serum total cholesterol (TC), triglycerides (TG), high-density lipoprotein (HDL), low-density lipoprotein (LDL), and very low-density lipoprotein (VLDL) were quantified spectrophotometrically utilizing a semi-automated biochemical analyzer (Erba Chem-7, Mannheim, Germany) in conjunction with commercial diagnostic kits (Egyptian Company for Biotechnology, Spectrum Diagnostics, Obour City, Cairo, Egypt) [20]. Serum urea concentration was determined colorimetrically using the Spectrum Diagnostics Urea/BUN–Liquizyme reagent (Egyptian Company for Biotechnology, Spectrum Diagnostics, Obour City, Cairo, Egypt; Ref. 318 001) according to the manufacturer’s instructions, employing the modified urease–Berthelot colorimetric method. In this assay, urea is hydrolyzed by urease to ammonia and carbon dioxide, and the liberated ammonia reacts under alkaline conditions with the chromogenic reagents to produce a colored complex proportional to the urea concentration. Serum creatinine concentration was determined using the Spectrum Diagnostics Creatinine–Jaffé reagent (Egyptian Company for Biotechnology, Spectrum Diagnostics, Obour City, Cairo, Egypt; Ref. 234 001), according to the manufacturer’s instructions, using the buffered kinetic Jaffé method without deproteinization. Creatinine reacts with picric acid under alkaline conditions to form a yellow-red complex, and the absorbance is proportional to the creatinine concentration. Concentrations of immunoglobulin G (IgG) and immunoglobulin M (IgM) were quantified utilizing commercial ELISA kits (Thermo Scientific, Waltham, MA, USA) in accordance with the manufacturer’s guidelines [6]. Biomarkers of oxidative stress, such as malondialdehyde (MDA), and superoxide dismutase (SOD) were assessed according to the methodologies outlined by Abd El-Hack et al. [21].

#### 2.2.4. Meat Quality

At the conclusion of the experimental period, samples of the *Longissimus lumborum* (LL) muscle were collected from the same seven rabbits per treatment immediately after slaughter and carcass processing. The proximate chemical composition of the LL muscle, including moisture, crude protein, crude fat, and ash contents, was determined according to the methods of the Association of Official Analytical Chemists (AOAC) [22]. The color parameters of uncooked LL muscle samples were assessed using a Minolta color reader CR-10 (Minolta Co. Ltd., Osaka, Japan) for the CIE color values: L* (lightness), a* (redness), and b* (yellowness) [23].

Physical meat quality characteristics were also evaluated. The meat pH in the LL muscle samples was determined 24 h postmortem by means of a portable calibrated digital pH meter, which is calibrated (using penetrating glass electrode; Hanna Instruments, Woonsocket, RI, USA). Three readings of meat pH were performed for each LL muscle sample, and the average reading was taken as final [24]. Also, the purge loss was calculated based on the method reported by Otto et al. [25]. In detail, around 50 g of LL muscle were weighed (initial weight), and each sample was separately packaged into polyethylene bags, kept at 4 °C for 48 h. Following storage, the samples were unpackaged, wiped dry with absorbent paper, and weighed again. Purge loss was calculated as follows:

Purge loss (%) = [(Initial weight − Final weight)/Initial weight] × 100

Water-holding capacity was measured based on filter paper press technique. About 0.3 g of ground LL muscle was placed between filter papers and subjected to compression force for 5 min at a constant pressure of 35 kg. The meat area and water area were measured, and the water holding capacity was calculated as percentage of retained water relative to the initial sample mass [26]. Cooking loss was determined according to the method described by Honikel [27]. Briefly, approximately 50 g of the Longissimus lumborum (LL) muscle was weighed and recorded as the initial weight. Each sample was individually placed in a heat-resistant polyethylene bag and cooked in a thermostatically controlled water bath maintained at 75 °C until the internal temperature of the meat reached 75 °C. The internal temperature was monitored by inserting a calibrated digital probe thermometer into the geometric center of each sample. The cooking process required approximately 1 h. After cooking, the samples were removed from the bags, cooled to room temperature, gently blotted with absorbent paper to remove surface moisture, and reweighed. Cooking loss was expressed as the percentage difference between the initial and cooked sample weights and calculated as follows:

Cooking loss (%) = [(Weight before cooking − Weight after cooking)/Weight before cooking] × 100

#### 2.2.5. Cecal Bacterial Populations

The cecum was removed aseptically from the same seven rabbits per treatment immediately after slaughter. Cecal contents were collected individually from each rabbit, placed into sterile containers, and immediately transported to the laboratory under refrigerated conditions (4 °C) for microbiological analysis. Cecal samples from each rabbit were processed individually. Ten grams from each sample were combined with 90 milliliters of sterile water pep-tone and incubated for thirty minutes. Liquid sample was collected to make a dilution of 10^−1^. Dilutions made were done at intervals of 10^−1^ to reach 10^−7^ dilution. Media were used to determine the concentrations of *Salmonella* spp., *Escherichia coli*, *Lactobacillus*, and total bacterial count [28].

For total bacterial count (TBC), appropriate dilutions were plated onto Plate Count Agar (PCA) and incubated aerobically at 37 °C for 48 h. *Lactobacillus* spp., were enumerated by plating on de Man, Rogosa, and Sharpe (MRS) agar followed by incubation at 37 °C for 48 h under anaerobic conditions. *Salmonella* spp. were enumerated on Xylose Lysine Deoxycholate (XLD) agar after aerobic incubation at 37 °C for 24 h. *Escherichia coli* was enumerated on Eosin Methylene Blue (EMB) agar under aerobic conditions at 37 °C for 24 h. For enumeration, 0.1 mL of each appropriate dilution was spread evenly onto the surface of the corresponding agar medium using a sterile spreader and plates containing countable colonies were selected for calculation. The number of colonies was multiplied by the corresponding dilution factor and corrected for the inoculated volume to determine the bacterial concentration. All microbial counts were expressed as log_10_ colony-forming units per gram (log_10_ CFU/g) of cecal content.

### 2.3. Statistical Assessment

Statistical analyses were performed using IBM SPSS Statistics for Windows, Version 26.0 (IBM Corp., Armonk, NY, USA) [29]. Before analysis, the data were examined for normality using the Shapiro–Wilk test and for homogeneity of variance using Levene’s test. Data that satisfied the assumptions of normality and homogeneity of variance were analyzed using a one-way analysis of variance (ANOVA) within the General Linear Model (GLM) procedure. The four dietary treatments (control, GA, MOS, and GA + MOS) were considered independent treatment groups, with dietary treatment included as the fixed effect. When a significant treatment effect was detected, Duncan’s multiple range test was used to compare treatment means [30]. Statistical significance was declared at *p* < 0.05. The statistical model used was:

Y_ij_ = μ + T_i_ + e_ij_

where e_ij_ is the random error related to the individual i_j_, T_i_ is the fixed influence of the ith treatment, μ is the overall mean, and Y_ij_ is the observation on the jth individual from the ith treatment.

## 3. Results

### 3.1. Growth Performance

**Figure 1:**
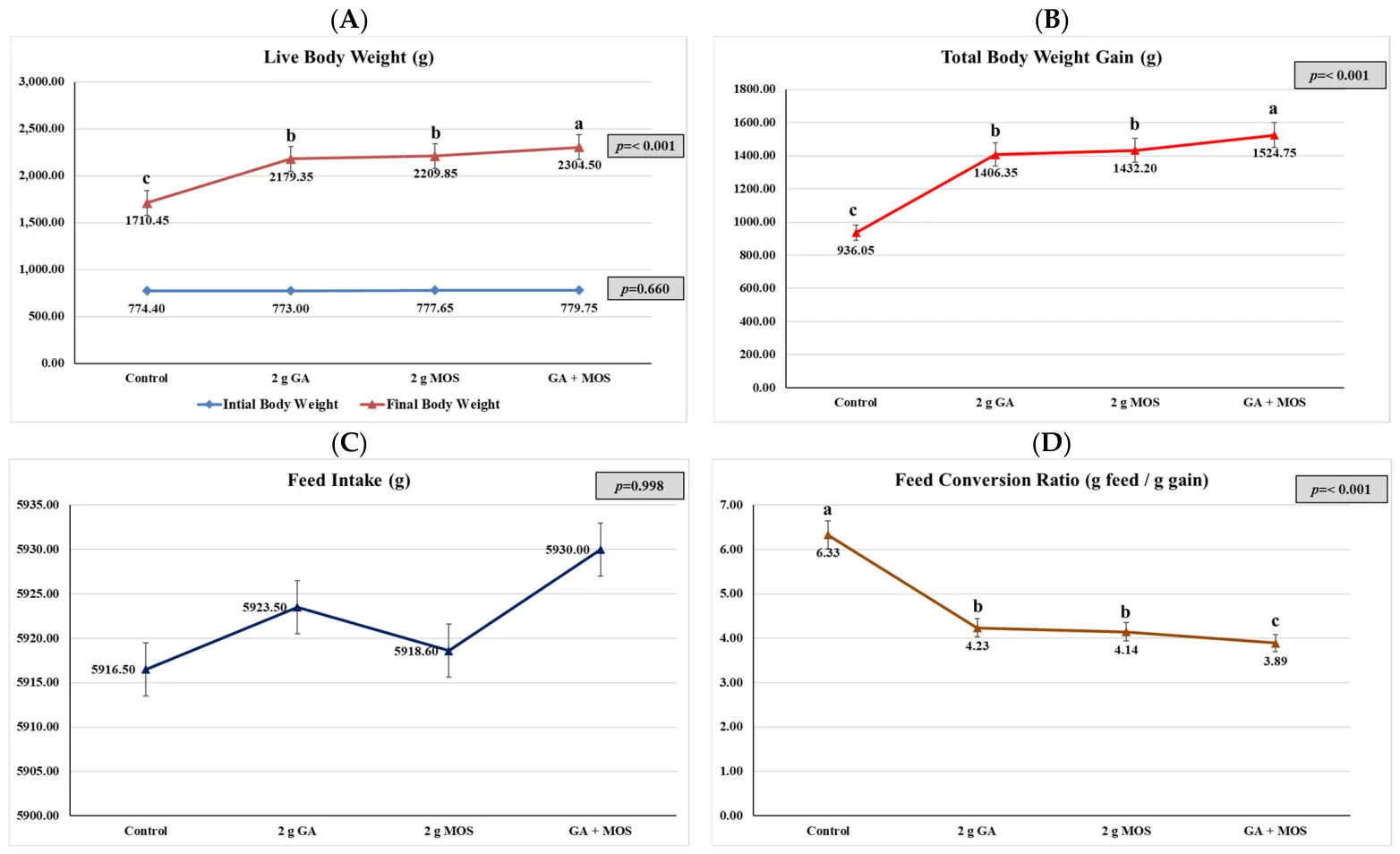
Effects of dietary gum Arabic (GA), mannan oligosaccharides (MOS), and their combination on growth performance of New Zealand White rabbits. (**A**) Live body weight (g); (**B**) total body weight gain (g); (**C**) feed intake (g); and (**D**) feed conversion ratio (g feed/g gain). Values are presented as means ± SE (*n* = 20 rabbits per treatment). Different superscript letters indicate significant differences among treatments (Duncan’s multiple range test, *p* < 0.05).

Figure 1 shows the effects of dietary supplementation of gum Arabic (GA), mannan oligosaccharides (MOS) and their combination on the growth performance of New Zealand White rabbits. There was no significant difference in initial body weight among the experimental groups (*p* = 0.660), which indicates similar starting point for all treatments. However, dietary supplementation significantly (*p* < 0.001) improved the final body weight (Figure 1A), total body weight gain (Figure 1B), and feed conversion ratio, but feed intake was not affected (*p* = 0.998); (Figure 1C).

The final body weight (2304.5 g) and total body weight gain (1524.75 g) were highest in rabbits fed with the combined supplementation of GA and MOS (*p* < 0.05) and were significantly higher than the groups receiving GA or MOS alone. The control rabbits had the lowest final body weight (1710.45 g) and body weight gain (936.05 g). Feed conversion ratio was significantly improved in all supplemented groups compared to the control. The FCR was most effective in the GA + MOS treatment (3.89) followed by MOS (4.14) and GA (4.23) while the control group showed the least efficient feed utilization (6.33) (Figure 1D).

### 3.2. Carcass Features

As shown in Table 2, dietary supplementation had a significant effect on most of carcass characteristics. Dietary supplementation affected pre-slaughter body weight and carcass yield (*p* < 0.001). The supplemented groups had greater pre-slaughter body weight and carcass yield than the control group. Although the GA + MOS group had the numerically highest values, it did not differ significantly from the GA and MOS groups for carcass yield.

Supplementation significantly decreased relative liver, kidney, giblets and abdominal fat percentages (*p* ≤ 0.01), but not heart percentage (*p* = 0.067). The lowest liver (2.99%), kidney (0.53%), giblets (3.85%) and abdominal fat (0.72%) percentages were recorded in rabbits fed GA + MOS, while the control rabbits recorded the highest values.

### 3.3. Blood Biochemical Parameters

The effect of dietary treatments on serum biochemical parameters is shown in Table 3. Dietary supplementation significantly increased (*p* < 0.001) the total protein and albumin concentration but did not affect the globulin concentration and albumin/globulin ratio (*p* > 0.05).

Dietary supplementation significantly decreased serum urea and creatinine concentrations and the activities of ALT and AST compared with the control group (*p* < 0.001). Although the GA + MOS group showed the numerically lowest urea concentration, it did not differ significantly from the GA and MOS groups.

Dietary supplementation also significantly altered the serum lipid profile. Total cholesterol, triglycerides, LDL, and VLDL concentrations were significantly lower in the supplemented groups (*p* < 0.001), whereas HDL concentration was significantly increased (*p* = 0.001) compared with the control group. Although the GA + MOS group had the numerically highest HDL concentration, it did not differ significantly from the GA group.

### 3.4. Antioxidant Status, Immunoglobulins

Table 4 shows the effects of dietary supplementation on serum antioxidant biomarkers and immune responses. Dietary supplementation significantly increased SOD activity and the concentrations of IgG and IgM, whereas MDA concentration was significantly decreased (*p* < 0.001). Serum SOD activity was higher in the supplemented groups than in the control group, with the highest numerical value observed in rabbits receiving the combined GA + MOS treatment (27.41 U/mL); however, the GA and MOS groups did not differ significantly from each other. In contrast, serum MDA concentration was significantly lower in the supplemented groups, with the lowest value recorded in the GA + MOS group (3.12 nmol/mL).

Similarly, serum IgG concentration was significantly higher in supplemented rabbits, reaching its highest value in the GA + MOS group (519.82 mg/dL). Serum IgM concentration was also significantly higher in all supplemented groups than in the control group.

### 3.5. Meat Quality Characteristics

The effects of dietary treatments on meat chemical composition, color attributes, and physical quality characteristics are summarized in Table 5. Dietary supplementation significantly affected several meat composition parameters. Moisture and crude protein contents increased (*p* < 0.001), whereas crude fat content decreased (*p* < 0.001). Ash percentage was also significantly affected by dietary treatment (*p* = 0.021), although the differences among the supplemented groups were relatively small.

Meat color parameters were also significantly affected by dietary supplementation. All supplemented groups showed higher lightness (L*), redness (a*), and yellowness (b*) values than the control group (*p* ≤ 0.006).

Dietary treatment also affected the physical characteristics of the meat. The GA + MOS group exhibited the highest ultimate pH and water-holding capacity (WHC), together with lower purge loss and cooking loss than the other groups (*p* < 0.01). The GA + MOS treatment recorded the highest WHC (70.63%) and the lowest purge loss (1.59%) and cooking loss (18.78%).

### 3.6. Cecal Bacterial Populations

The effect of dietary supplementation on the cecal bacterial population is shown in Table 6. Dietary treatment did not significantly affect the total bacterial count (*p* = 0.058), although the counts tended to be lower in the supplemented groups. Similarly, *Salmonella* spp. counts were numerically lower in the supplemented groups, but the differences were not statistically significant (*p* = 0.088).

In contrast, *Lactobacillus* spp. counts were significantly increased by dietary supplementation (*p* < 0.001), with the highest population observed in the GA + MOS group. Cecal *E. coli* counts differed significantly among treatments (*p* = 0.004); however, the control, GA, and MOS groups did not differ significantly from one another. The significantly lower *E. coli* count was observed only in rabbits receiving the combined GA + MOS treatment compared with the other treatment groups.

## 4. Discussion

The present study showed that dietary supplementation with GA, MOS, and their combined treatment affected several growth-performance parameters in New Zealand White rabbits. Final body weight and total body weight gain were highest in the GA + MOS group, while feed conversion ratio was improved in all supplemented groups compared with the control. In contrast, feed intake was not significantly affected by dietary treatment. Thus, the observed differences in body weight gain and feed conversion ratio occurred without a corresponding increase in feed intake.

Addition of gum Arabic to the diet has previously been associated with positive effects on growth performance, which have been attributed to changes in intestinal microbial fermentation, antioxidant status, and nutrient utilization [9,31]. Similarly, previous studies have reported that MOS supplementation can improve body weight gain and feed conversion ratio, with proposed explanations involving modulation of the intestinal microbiota, intestinal development, and immune responses [32,33]. These mechanisms provide a possible biological context for the responses observed in the present study. Previous studies have also examined combinations of different prebiotic compounds and reported favorable productive responses [34], which is consistent with the present observation that the combined GA + MOS treatment performed favorably.

The favorable growth response observed with GA supplementation may be related, at least in part, to the known characteristics of GA as a fermentable soluble fiber. Gum Arabic consists largely of arabinogalactan polysaccharides that are relatively resistant to digestion in the upper gastrointestinal tract and can subsequently be fermented by microorganisms in the hindgut [35]. Fermentation of GA has been reported to generate SCFAs, including acetate, propionate, and butyrate [36]. These metabolites have recognized roles in intestinal physiology and may contribute to epithelial energy supply and gut barrier maintenance. However, SCFA concentrations were not determined in the present study; therefore, enhanced SCFA production cannot be confirmed as the mechanism responsible for the growth responses observed here. SCFAs have also been reported to influence intestinal pH and microbial populations [37]. Accordingly, these effects may provide one possible explanation for the performance responses to GA, but measurements of cecal SCFAs and pH would be required to establish this relationship experimentally.

Mannan oligosaccharides work through different mechanisms that may complement those proposed for fermentable dietary fibers. MOS can act as a mannose-containing binding substrate for pathogenic bacteria expressing type-1 fimbriae, including *Escherichia coli* and *Salmonella* spp., thereby interfering with their attachment to intestinal epithelial cells [38]. This proposed mechanism is relevant to the microbial findings of the present study, particularly because the GA + MOS treatment had a significantly lower *E. coli* count than the other treatments. However, bacterial adhesion to intestinal epithelial cells was not directly assessed, and therefore inhibition of pathogen adhesion cannot be confirmed from the present data. MOS has also been associated with changes in beneficial bacterial populations and immune responses in previous studies [39,40]. In the present experiment, an increase in cecal *Lactobacillus* spp. was observed, particularly in the GA + MOS group.

The present study showed that dietary supplementation with GA, MOS, and particularly the combined treatment affected several carcass characteristics of NZW rabbits. Pre-slaughter weight and carcass yield were increased by dietary supplementation, with the highest numerical values observed in the GA + MOS group. Relative liver, kidney, giblet, and abdominal fat percentages were also lower in the supplemented rabbits, whereas heart percentage was not significantly affected. These findings indicate that the combined dietary treatment was associated with favorable carcass responses. The present results are in agreement with previous reports showing that dietary supplementation with gum Arabic or MOS can affect carcass yield and abdominal fat deposition in rabbits and other monogastric animals [41,42]. The higher carcass yield observed in the supplemented groups may be related to the greater body weight and improved feed conversion ratio observed in the present study [43]. However, because nutrient digestibility and body-composition partitioning were not directly measured, the precise mechanism underlying these carcass responses remains uncertain.

Prebiotics, including GA and MOS, have been reported to influence intestinal microbial populations and intestinal function, with potential consequences for nutrient digestion and absorption [33,44]. These findings from previous studies provide a plausible background for interpreting the present carcass responses, but they should not be taken as direct evidence that improved nutrient digestibility or intestinal barrier function occurred in the current experiment.

The lower abdominal fat percentage observed in the supplemented rabbits may reflect differences in nutrient partitioning associated with dietary supplementation; however, the present study did not directly measure lipid synthesis, fatty acid oxidation, or the activity of lipogenic enzymes. Gum Arabic can be fermented by cecal microorganisms to produce SCFAs, particularly propionate, and previous studies have proposed that SCFAs can influence hepatic lipid metabolism through effects on lipogenic pathways [45,46]. These observations provide a possible explanation for the lower abdominal fat observed in the supplemented groups, but neither SCFA production nor hepatic lipogenesis was measured in the present study. Similarly, SCFAs have been reported to influence AMPK, a regulator of cellular energy metabolism [47]. MOS has also been reported to influence intestinal microbial populations and metabolic responses [48]. Thus, changes in microbial composition may potentially contribute to differences in fat deposition, but this possibility requires direct investigation through measurements of microbial metabolites and lipid-metabolism pathways.

The lower relative liver and kidney weights observed in the supplemented groups should also be interpreted cautiously. Although organ weights can provide useful information about physiological responses to dietary treatments, they do not by themselves demonstrate improved liver or kidney function. The present study measured serum biochemical indicators of hepatic and renal status, and the observed changes should therefore be discussed primarily as changes in these biochemical parameters rather than as direct evidence of altered organ function. Histopathological examination and additional functional assessments would be required to determine whether the differences in relative organ weights represent changes in organ health or metabolic activity [49].

The present study showed that dietary supplementation with GA, MOS, and their combined treatment significantly affected several serum biochemical parameters in New Zealand White rabbits. The combined GA + MOS treatment generally produced the most favorable responses, including higher serum total protein and albumin concentrations and lower serum urea, creatinine, ALT, and AST concentrations compared with the control. However, these changes should be interpreted as alterations in serum biochemical indices rather than direct evidence of improved hepatic or renal function.

The increased serum total protein and albumin concentrations observed in the supplemented groups may reflect differences in protein status associated with dietary supplementation. Albumin is synthesized primarily by hepatocytes and is commonly used as an indicator of protein status and hepatic synthetic function [50]. However, the present study did not measure amino acid availability, nutrient digestibility, intestinal absorption, or hepatic protein synthesis. Therefore, it would be inappropriate to conclude that GA or MOS increased amino acid availability or nutrient utilization. The observed increases in total protein and albumin may instead indicate a favorable change in the measured serum protein profile, while the precise mechanisms responsible require further investigation [51].

The lower serum ALT and AST activities observed in the supplemented rabbits are consistent with differences in these biochemical indicators of hepatic status. Increased circulating activities of these enzymes can occur with hepatocellular damage or altered cellular membrane permeability [52]. Previous studies have proposed several mechanisms through which GA may influence hepatic biochemical responses. For example, GA fermentation can generate SCFAs, including butyrate and propionate, which have been associated with anti-inflammatory and antioxidant effects [53,54]. GA has also been reported to contain bioactive components that may contribute to antioxidant activity [55].

MOS may also contribute to changes in serum biochemical profiles through its previously reported effects on intestinal microbial populations and pathogen adhesion [56].

The reductions in serum urea and creatinine concentrations observed in the supplemented groups indicate changes in these two measured indicators of renal and protein metabolism [57]. However, lower serum urea and creatinine concentrations do not by themselves demonstrate improved glomerular filtration or enhanced renal function, particularly in healthy animals. Therefore, the lower concentrations should be regarded as favorable changes in the measured biochemical indices, while their precise physiological significance requires further investigation.

Dietary supplementation also affected several serum lipid parameters in the present study. The observed changes in cholesterol, triglycerides, and related lipid indices suggest that GA and MOS influenced the measured serum lipid profile. Fermentation of GA has been reported to generate propionate, which may influence cholesterol metabolism and hepatic lipid synthesis through mechanisms involving HMG-CoA reductase [15]. Likewise, SCFAs have been reported to interact with metabolic signaling pathways, including AMPK [16]. MOS has also been reported to influence lipid metabolism through changes in intestinal microbial populations and bile acid metabolism [58].

Overall, the serum biochemical findings suggest that GA, MOS, and their combined dietary treatment were associated with favorable changes in several measured indicators of protein, lipid, hepatic, and renal status. The generally favorable responses observed with GA + MOS may support the practical use of the two supplements together. The different biological properties reported for GA and MOS may provide a plausible explanation for the favorable response of the combined treatment, but confirmation of these mechanisms would require targeted measurements in future studies. Previous studies have reported that dietary GA supplementation can affect liver and kidney-related biochemical parameters, protein metabolism, and serum lipids in rabbits and other monogastric animals, potentially in association with its antioxidant and prebiotic properties [9,41]. Similarly, previous research has reported effects of MOS supplementation on serum cholesterol and triglycerides and on indicators related to physiological and metabolic status [59].

The present study also demonstrated significant effects of dietary supplementation on selected antioxidant indices. The supplemented rabbits showed higher SOD activity and lower serum MDA concentrations than the control group, indicating a favorable change in the measured antioxidant status. Oxidative stress results from an imbalance between reactive oxygen species (ROS) generation and antioxidant defense and can lead to oxidative damage to cellular components [60]. SOD is an important antioxidant enzyme that converts superoxide radicals into hydrogen peroxide, which can subsequently be metabolized by other antioxidant enzymes, including catalase and glutathione peroxidase [61]. Accordingly, the higher SOD activity observed in the supplemented groups indicates an enhanced activity of this measured antioxidant defense component. Similarly, MDA is commonly used as an indicator of lipid peroxidation [62], and its lower concentration in the supplemented groups suggests reduced lipid peroxidation under the conditions of the present experiment.

Several mechanisms have previously been proposed to explain the antioxidant effects of GA. Its fermentation by intestinal microorganisms can generate SCFAs, and previous studies have suggested that these metabolites may influence cellular antioxidant pathways, including Nrf2 signaling [63]. GA-associated bioactive components have also been proposed to contribute to free-radical-scavenging activity [64]. MOS may affect antioxidant status indirectly through its reported effects on intestinal microbial populations and pathogen-associated responses [65]. Previous studies have also suggested that MOS can support populations of beneficial microorganisms such as *Lactobacillus* spp., which may contribute to intestinal and systemic physiological responses [66].

The present study further demonstrated that GA, MOS, and their combined treatment affected humoral immune indicators, with higher serum IgG and IgM concentrations observed in the supplemented rabbits. Immunoglobulins are important components of adaptive immunity and participate in pathogen recognition and neutralization. Thus, the higher IgG and IgM concentrations indicate a favorable change in the measured humoral immune response. Previous studies have reported immunomodulatory effects of GA that may be associated with fermentation-derived SCFAs and arabinogalactan polysaccharides [67,68]. SCFAs, particularly butyrate, have been reported to influence immune-cell differentiation and inflammatory responses, while arabinogalactan-containing compounds have been investigated for their effects on immune-cell activity [67,68]. Similarly, MOS has been reported to influence immune responses and gut-associated lymphoid tissue (GALT) through several proposed mechanisms [69].

The improved serum antioxidant and immunoglobulin responses observed with the combined GA + MOS treatment indicate that this dietary regimen was associated with favorable changes in the measured antioxidant and humoral immune parameters. The different biological properties reported for GA and MOS may provide a plausible basis for the response observed with their combined inclusion. Instead, GA and MOS may provide complementary dietary effects, a possibility that should be investigated further using targeted measurements of SCFAs, intestinal morphology and barrier function, inflammatory mediators, microbial metabolites, and relevant molecular signaling pathways. The present findings are generally consistent with previous reports that dietary GA or MOS supplementation can influence antioxidant enzyme activities, lipid peroxidation, and humoral immune responses in rabbits, poultry, and other monogastric species [11,41,70,71].

The present study showed that dietary supplementation with GA, MOS, and their combined treatment affected several nutritional and physicochemical characteristics of rabbit meat. The supplemented groups showed higher crude protein and lower crude fat contents, with the most favorable numerical responses generally observed in the GA + MOS treatment. These findings indicate differences in the proximate composition of meat among dietary treatments. Previous studies have reported associations between dietary supplementation and changes in meat composition [72,73], providing a possible context for the present findings.

The lower crude fat content observed in supplemented rabbits may also be related to differences in lipid deposition. Previous research has suggested that fermentation products of GA may influence lipid metabolism through pathways involving lipogenic enzymes and AMPK [74]. Likewise, MOS has been reported to affect intestinal microbial populations and metabolic responses [75]. These mechanisms may provide possible explanations for the differences in meat fat content observed in the present study.

Dietary supplementation also resulted in significant changes in instrumental meat-color characteristics. The supplemented rabbits showed higher values of lightness (*L*), redness (*a*), and yellowness (*b*) than the control group. These changes indicate that dietary treatment influenced the instrumental color properties of rabbit meat. Meat color is influenced by muscle pigments, their chemical state, and postmortem biochemical changes. Previous studies have suggested that antioxidant status may contribute to the stability of myoglobin and meat color [76], while reduced lipid oxidation may also help preserve muscle pigments [77]. Thus, the improved instrumental color values observed in the supplemented groups may be associated with differences in oxidative status.

The increase in water-holding capacity (WHC) and the reductions in purge loss and cooking loss observed in supplemented rabbits indicate improved water retention properties of the meat. These are directly measured physicochemical characteristics and therefore provide evidence of differences in meat-processing quality among dietary treatments. Previous studies have associated WHC and cooking loss with muscle pH, protein denaturation, and the physicochemical properties of muscle proteins [78,79]. Similarly, although previous research has suggested relationships between oxidative status, hepatic condition, pre-slaughter stress, and postmortem muscle changes [80], pre-slaughter stress was not assessed in the present study through cortisol concentrations, behavioral indicators, or other physiological stress markers.

The favorable meat-quality responses observed with GA and MOS may be related to their previously reported effects on intestinal and metabolic physiology. GA-derived SCFAs have been proposed to influence antioxidant status and cellular metabolism, whereas MOS has been associated with modulation of intestinal microbial populations and immune responses [11]. The different biological properties reported for GA and MOS provide a plausible background for future investigations into how these additives may influence meat quality. Previous studies have reported that prebiotic supplementation can improve WHC, reduce cooking loss, and affect other indicators of meat quality [81,82,83], which provides supporting context for the present observations.

The present study also demonstrated changes in selected culturable cecal bacterial populations. In particular, the GA + MOS treatment showed a higher *Lactobacillus* spp. count and a significantly lower *Escherichia coli* count than the other dietary treatments. Importantly, the control, GA, and MOS groups did not differ significantly in *E. coli* count. Therefore, the reduction in *E. coli* should be attributed specifically to the combined GA + MOS treatment rather than to GA or MOS supplementation individually.

The higher *Lactobacillus* spp. count and lower *E. coli* count observed in the GA + MOS group indicate a favorable change in the selected culturable cecal bacterial populations measured in this study. GA is a fermentable dietary substrate that has been reported to support microbial fermentation and SCFA production [35], whereas MOS can interfere with the attachment of bacteria expressing mannose-specific fimbriae [17]. These previously reported properties provide plausible explanations for the microbial responses observed here.

The increased *Lactobacillus* spp. count may be relevant to the favorable growth, physiological, and meat-quality responses observed in the supplemented rabbits, because beneficial intestinal bacteria have previously been associated with host nutrition and immune function [84]. The lower *E. coli* count in the GA + MOS group should be interpreted as a measured microbiological response rather than direct evidence of reduced intestinal inflammation, improved barrier function, or enhanced nutrient utilization. MOS has previously been reported to reduce pathogen colonization [36,85], which provides a possible explanation for the present observation.

Previous studies have reported favorable effects of combinations of prebiotic compounds on intestinal microbial populations and animal performance [86]. The present findings are broadly consistent with this literature because the combined GA + MOS treatment showed favorable responses in selected cecal bacterial populations and several productive and meat-quality traits.

While this study provides valuable insights into the effects of gum Arabic and mannan oligosaccharides in growing rabbits, several limitations should be acknowledged. The cecal microbial analysis relied on culture-dependent methods targeting only a few bacterial groups (*Lactobacillus*, *E. coli*, and *Salmonella*), which does not capture the full complexity, diversity, or functional capacity of the gut microbiome. Advanced techniques such as 16S rRNA sequencing would be needed for a more comprehensive assessment. Also, the biological mechanisms proposed to explain the observed responses—including enhanced nutrient utilization, improved intestinal morphology, reduced inflammation, and activation of signaling pathways such as Nrf2 and AMPK—were not directly measured in this study and remain speculative. Moreover, organ function was inferred from serum biochemical parameters and relative organ weights, but histopathological examination or functional biomarkers would be required to confirm these interpretations.

## 5. Conclusions

Supplementation with gum Arabic (GA) and mannan oligosaccharides (MOS) enhanced certain facets of productive performance, physiological condition, meat quality, and cecal microbial communities in developing New Zealand White rabbits. The singular and collective therapies yielded positive outcomes for several assessed parameters; yet, the extent and statistical relevance of these benefits differed between features. While the GA + MOS therapy had the most advantageous numerical outcomes for certain parameters, it did not consistently outperform the separate therapies. The recorded reactions were linked to alterations in antioxidant levels, specific immune-related markers, and cecal microbial communities, whereas nutrition consumption was not directly assessed and should not be interpreted as a mechanism for the observed reactions. In summary, both GA and MOS, whether used separately or together, seem to be effective dietary supplements for enhancing specific productive and physiological outcomes in growing rabbits within the context of this study.

## Figures and Tables

**Table 1:** Ingredients and calculated chemical composition of the basal diet.

Ingredients	(%)
Alfalfa hay	26.5
Barley	17.0
Yellow corn	15.0
Soybean meal (44%)	16.0
Wheat bran	20.0
Alfalfa straw	3.0
Limestone	1.65
Vitamins & minerals premix *	0.30
NaCl	0.30
dl- methionine	0.1
Anti-coccidia	0.05
Anti-toxin	0.1
Total	100.0
Chemical composition (as DM basics).	
Crude protein	17.5
Fat	2.8
Digestible energy (kcal/kg)	2600
Crude fiber	10.0
Calcium	0.93
Total phosphorus	0.62
Lysine	0.84
Methionine	0.38
Methionine + Cysteine	0.69

* Supplied per 1 kg diet: 6000 IU vit. A; 900 IU vit. D3; 40 mg vit. E; 2.0 mg vit. K3; 2.0 mg vit. B1; 4.0 mg vit. B2; 2.0 mg vit. B6; 0.010 mg vit. B12; 5.0 mg vit. PP; 10.0 mg vit. B5; 0.05 mg B8; 3.0 mg B9; 250 mg choline; 50.0 mg Fe; 50.0 mg Zn; 8.5 mg Mn; 5.0 mg Cu; 0.20 mg I, and 40.0 mg Se.

**Table 2:** Influences of dietary supplementation with gum Arabic (GA), mannan oligosaccharides (MOS) and its combination on carcass traits of New Zealand White (NZW) Rabbits.

Traits	Treatments (g/kg Diet)				*p* Value
	Control	2 g GA	2 g MOS	GA + MOS	
Pre-slaughtered weight	1781.25 ± 10.87 ^c^	2107.5 ± 12.52 ^b^	2108.75 ± 15.47 ^b^	2275.00 ± 10.16 ^a^	<0.001
Carcass %	43.17 ± 0.706 ^b^	50.99 ± 1.62 ^a^	51.40 ± 0.810 ^a^	52.30 ± 0.514 ^a^	<0.001
Liver %	3.80 ± 0.167 ^a^	3.20 ± 0.159 ^b^	3.34 ± 0.111 ^b^	2.99 ± 0.108 ^b^	0.010
Heart %	0.39 ± 0.034	0.31 ± 0.017	0.34 ± 0.011	0.31 ± 0.009	0.067
Kidney %	0.74 ± 0.026 ^a^	0.60 ± 0.003 ^b^	0.60 ± 0.018 ^b^	0.53 ± 0.005 ^c^	<0.001
Giblets %	4.94 ± 0.158 ^a^	4.12 ± 0.166 ^b^	4.29 ± 0.132 ^b^	3.85 ± 0.118 ^b^	0.001
Abdominal fat %	1.29 ± 0.024 ^a^	0.94 ± 0.021 ^b^	0.94 ± 0.009 ^b^	0.72 ± 0.016 ^c^	<0.001

^a–c^ Means within the same row with different superscripts are significantly different (Duncan’s multiple range test, *p* < 0.05). Values are presented as means ± SE (*n* = 7 rabbits per treatment).

**Table 3:** Influences of dietary supplementation with gum Arabic (GA), mannan oligosaccharides (MOS) and its combination on blood biochemical parameters of New Zealand White (NZW) Rabbits.

Traits	Treatments (g/kg Diet)				*p* Value
	Control	2 g GA	2 g MOS	GA + MOS	
**Liver and kidney functions**					
TP (g/dL)	4.50 ± 0.193 ^c^	5.53 ± 0.201 ^b^	5.53 ± 0.112 ^b^	6.80 ± 0.053 ^a^	<0.001
ALB (g/dL)	3.17 ± 0.095 ^c^	4.13 ± 0.253 ^b^	4.27 ± 0.210 ^b^	5.25 ± 0.035 ^a^	<0.001
GLOB (g/dL)	1.32 ± 0.126	1.40 ± 0.245	1.25 ± 0.129	1.54 ± 0.067	0.597
A/G ratio	2.46 ± 0.224	3.37 ± 0.891	3.55 ± 0.479	3.41 ± 0.161	0.465
ALT (IU/L)	50.01 ± 2.18 ^a^	43.54 ± 0.862 ^b^	41.06 ± 0.534 ^b^	36.64 ± 0.914 ^c^	<0.001
AST (IU/L)	112.89 ± 0.820 ^a^	107.69 ± 1.06 ^b^	108.71 ± 1.33 ^b^	100.73 ± 0.481 ^c^	<0.001
Urea (mg/dL)	42.65 ± 0.472 ^a^	34.25 ± 1.03 ^b^	32.42 ± 1.36 ^b^	30.62 ± 1.55 ^b^	<0.001
Creatinine (mg/dL)	0.84 ± 0.012 ^a^	0.74 ± 0.012 ^b^	0.70 ± 0.011 ^c^	0.63 ± 0.011 ^d^	<0.001
**Lipid profile**					
TC (mg/dL)	109.52 ± 3.061 ^a^	89.81 ± 0.380 ^b^	87.43 ± 0.850 ^b^	77.99 ± 0.951 ^c^	<0.001
TG (mg/dL)	90.85 ± 0.565 ^a^	77.73 ± 0.470 ^b^	76.82 ± 0.792 ^b^	68.48 ± 0.723 ^c^	<0.001
HDL (mg/dL)	31.73 ± 1.13 ^c^	38.84 ± 1.52 ^ab^	37.11 ± 1.10 ^b^	41.10 ± 1.03 ^a^	0.001
LDL (mg/dL)	59.62 ± 4.05 ^a^	35.42 ± 1.36 ^b^	34.95 ± 1.82 ^b^	23.19 ± 1.01 ^c^	<0.001
VLDL (mg/dL)	18.17 ± 0.113 ^a^	15.54 ± 0.094 ^b^	15.36 ± 0.158 ^b^	13.69 ± 0.144 ^c^	<0.001

^a–d^ Means within the same row with different superscripts are significantly different (Duncan’s multiple range test, *p* < 0.05). Values are presented as means ± SE (*n* = 7 rabbits per treatment). TP: total protein; ALB: albumin; GLOB: globulin; AST: aspartate aminotransferase; ALT: alanine aminotransferase; TC: total cholesterol; TG: triglycerides; HDL: high-density lipoprotein; LDL: low-density lipoprotein, and VLDL: very low-density lipoprotein.

**Table 4:** Influences of dietary supplementation with gum Arabic (GA), mannan oligosaccharides (MOS) and its combination on blood antioxidant parameters and immunoglobulin levels of New Zealand White (NZW) Rabbits.

Traits	Treatments (g/kg Diet)				*p* Value
	Control	2 g GA	2 g MOS	GA + MOS	
**Antioxidant parameters**					
SOD (U/mL)	16.84 ± 0.489 ^c^	22.74 ± 0.531 ^b^	22.85 ± 0.494 ^b^	27.41 ± 0.949 ^a^	<0.001
MDA (nmol/mL)	4.24 ± 0.155 ^a^	3.67 ± 0.031 ^b^	3.40 ± 0.020 ^c^	3.12 ± 0.021 ^d^	<0.001
**Immunoglobulin levels**					
IgG (mg/dL)	452.29 ± 1.42 ^c^	483.19 ± 1.82 ^b^	490.93 ± 1.66 ^b^	519.82 ± 1.71 ^a^	<0.001
IgM (mg/dL)	105.97 ± 3.79 ^b^	136.24 ± 1.28 ^a^	137.82 ± 0.545 ^a^	141.11 ± 0.659 ^a^	<0.001

^a–d^ Means within the same row with different superscripts are significantly different (Duncan’s multiple range test, *p* < 0.05). Values are presented as means ± SE (*n* = 7 rabbits per treatment). SOD: superoxide dismutase; MDA: Malondialdehyde; IgG: immunoglobulin G; and IgM: immunoglobulin M.

**Table 5:** Influences of dietary supplementation with gum Arabic (GA), mannan oligosaccharides (MOS) and its combination on meat quality parameters of New Zealand White (NZW) Rabbits.

Traits	Treatments (g/kg Diet)				*p* Value
	Control	2 g GA	2 g MOS	GA + MOS	
**Chemical composition analysis (%)**					
Moisture content	68.67 ± 0.427 ^c^	70.35 ± 0.374 ^b^	71.06 ± 0.360 ^ab^	71.90 ± 0.252 ^a^	<0.001
Crude protein	21.21 ± 0.362 ^b^	22.80 ± 0.05 ^a^	22.75 ± 0.175 ^a^	23.05 ± 0.146 ^a^	<0.001
Crude fat	3.85 ± 0.088 ^a^	3.17 ± 0.041 ^b^	3.08 ± 0.008 ^bc^	2.97 ± 0.036 ^c^	<0.001
Ash	1.25 ± 0.059 ^ab^	1.33 ± 0.022 ^a^	1.30 ± 0.028 ^a^	1.15 ± 0.024 ^b^	0.021
**Color measurements**					
L*	51.73 ± 0.602 ^b^	59.90 ± 0.738 ^a^	61.15 ± 0.272 ^a^	61.27 ± 0.691 ^a^	<0.001
a*	10.47 ± 0.303 ^b^	15.05 ± 1.309 ^a^	14.67 ± 0.438 ^a^	16.80 ± 0.674 ^a^	0.001
b*	11.20 ± 0.351 ^b^	12.79 ± 0.592 ^a^	13.62 ± 0.160 ^a^	13.12 ± 0.375 ^a^	0.006
**Physical meat quality characteristics**					
pH	5.37 ± 0.131 ^b^	5.52 ± 0.047 ^b^	5.57 ± 0.062 ^b^	5.87 ± 0.025 ^a^	0.005
WHC %	55.74 ± 0.664 ^c^	64.74 ± 0.253 ^b^	65.36 ± 0.208 ^b^	70.63 ± 0.29 ^a^	<0.001
Purge loss %	3.90 ± 0.059 ^a^	2.43 ± 0.188 ^b^	2.4 ± 0.146 ^b^	1.59 ± 0.069 ^c^	<0.001
Cooking loss	30.13 ± 1.07 ^a^	23.04 ± 0.839 ^b^	21.5 ± 0.361 ^b^	18.78 ± 0.321 ^c^	<0.001

^a–c^ Means within the same row with different superscripts are significantly different (Duncan’s multiple range test, *p* < 0.05). Values are presented as means ± SE (*n* = 7 rabbits per treatment). WHC: Water-holding capacity; L*: Lightness; a*: Redness/Greenness; b*: Yellowness/Blueness.

**Table 6:** Influences of dietary supplementation with gum Arabic (GA), mannan oligosaccharides (MOS) and its combination on cecal bacterial count (log10 CFU/g cecal content) of New Zealand White (NZW) Rabbits.

Traits	Treatments (g/kg Diet)				*p* Value
	Control	2 g GA	2 g MOS	GA + MOS	
TBC	10.44 ± 0.006	10.22 ± 0.007	10.19 ± 0.009	10.14 ± 0.008	0.058
*Lactobacillus* spp.	6.99 ± 0.04 ^c^	7.48 ± 0.033 ^b^	7.53 ± 0.011 ^b^	7.65 ± 0.011 ^a^	<0.001
*Salmonella* spp.	3.95 ± 0.032	3.59 ± 0.045	3.42 ± 0.171	2.36 ± 0.797	0.088
*E. coli*	4.48 ± 0.04 ^a^	3.76 ± 0.048 ^a^	3.65 ± 0.073 ^a^	1.57 ± 0.911 ^b^	0.004

^a–c^ Means within the same row with different superscripts are significantly different (Duncan’s multiple range test, *p* < 0.05). Values are presented as means ± SE (*n* = 7 rabbits per treatment). TBC: Total bacterial count.

## Data Availability

The data presented in this study are available from the corresponding author upon reasonable request. Data are not publicly available because they are subject to ethical and privacy restrictions associated with the animal study.

## Abbreviations

The following abbreviations are used in this manuscript:

GA	Gum Arabic
MOS	Mannan Oligosaccharides
NZW	New Zealand White
SCFAs	Short-Chain Fatty Acids
Nrf2	Nuclear Factor Erythroid 2-Related Factor 2
NF-κB	Nuclear Factor-Kappa B
GALT	Gut-Associated Lymphoid Tissue
FCR	Feed Conversion Ratio
IgG	Immunoglobulin G
IgM	Immunoglobulin M
SOD	Superoxide dismutase
MDA	Malondialdehyde
ALT	Alanine aminotransferase
AST	Aspartate aminotransferase
WHC	Water-Holding Capacity
ROS	Reactive Oxygen Species
